# Catechin (epigallocatechin-3-gallate) supplement restores the oxidation: antioxidation balance through enhancing the total antioxidant capacity in Wistar rats with cadmium-induced oxidative stress

**DOI:** 10.1017/jns.2025.10040

**Published:** 2025-09-19

**Authors:** Mohammed Al-Zharani, Mohammed Mubarak, Eman Almuqri, Hassan Rudayni, Nada Aljarba, Khadijah Yaseen, Shaikha Albatli, Saad Alkahtani, Fahd Nasr, Amin Al-Doaiss, Mohammed Al-eissa

**Affiliations:** 1 Department of Biology, College of Science, Imam Mohammad Ibn Saud Islamic University (IMSIU), Riyadh, Saudi Arabia; 2 Department of Biology, College of science, Princess Nourah bint Abdulrahman University, Riyadh 11671, Saudi Arabia; 3 Department of Zoology, College of Science, King Saud University, Riyadh, Saudi Arabia; 4 Department of Biology, College of Science, King Khalid University, Abha, Saudi Arabia

**Keywords:** Antioxidant, Biochemical profile, Cadmium toxicity, Catechin, Oxidative stress

## Abstract

Catechins are bioactive flavanols commonly found in the fruits and leaves of plants, particularly the fresh tea leaves. This experimental study aims to evaluate the antioxidant properties of epigallocatechin-3-gallate, one of the most prominent catechins, and its ability to mitigate cadmium-induced oxidative stress. Eighty rats were randomly assigned to four groups of 20: an untreated control group (group 1), a catechin-treated group (group 2), a cadmium-exposed group (group 3), and a cadmium-catechin group (group 4). Group 2 rats received daily oral doses of catechin at 300 mg/kg body weight, while Group 3 rats were given an aqueous solution of cadmium chloride at a final concentration of 5 mg/kg body weight (b.w.) per day. Group 4 rats were treated with both catechin and cadmium chloride. The rats in Group 4 exhibited increased levels of total proteins and significant increases in antioxidant markers, including total thiols, glutathione, total antioxidant capacity, superoxide dismutase, glutathione peroxidase, and catalase. Additionally, this group demonstrated significant decreases in blood cadmium levels and in the following enzymes: alkaline phosphatase, alanine aminotransferase, and aspartate aminotransferase. They also demonstrated significant decreases in creatinine, blood urea nitrogen, urea, and bilirubin, as well as in oxidation markers (H_2_O_2_ and malondialdehyde), compared to the cadmium group (Group 3). Tissue homogenates from the livers and kidneys of Group 4 rats revealed similar results to those of the serum biochemical assay. Based on these findings, it can be concluded that catechin’s (ECGC) antioxidant properties significantly mitigate cadmium-induced oxidative stress.

## Graphical abstract

**Figure:**
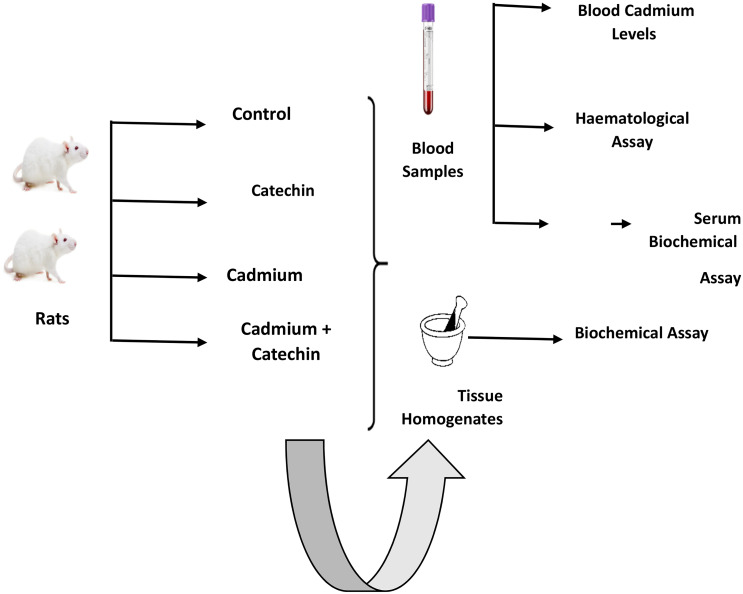

## Introduction

Catechins are bioactive phytochemicals (flavanols) commonly found in the fruits and leaves of plants, including tea leaves, peaches, strawberries, raspberries, blackberries, and cocoa beans. The major polyphenols found in green tea are epicatechin, epicatechin gallate, epigallocatechin, and epigallocatechin gallate.^(1–3)^ The most abundant catechin in green tea is epigallocatechin-3-gallate (EGCG).

Conducted research has demonstrated a wide range of health benefits and protective effects of catechins, as shown in clinical and experimental investigations focusing on cardiovascular diseases, diabetes, cancer, and neurodegenerative diseases.^(4–6)^ The chemical structure of catechins generally determines their potential bioactivities. Among flavonol catechins, EGCG (C_22_H_18_O_11_), a polyphenolic gallate ester, is the most health-promoting constituent in green tea due to its potent antitumour, anti-inflammatory, and antioxidant properties^(6–8)^.

Oxidative stress is a series of events that leads to an imbalance between reactive oxygen species (ROS) activity and the efficacy of the endogenous antioxidant system in counteracting the harmful effects of ROS.^(9)^ Oxidation reactions, triggered by certain metabolites or external toxins, contribute to various forms of cellular damage. These effects can be severe enough to lead to cell death and, in turn, significant tissue damage. The excessive generation rate of ROS results in oxidative stress, which exacerbates oxidation-induced tissue damage.^(10)^

Excessive ROS generation can damage cellular lipids, proteins, and DNA, causing neurodegenerative disorders, cardiovascular diseases, cancer, diabetes, accelerated ageing, and immune dysfunction.^(11,12)^ In other words, oxidative stress could significantly contribute to the development of medically concerning complications. These effects can be counteracted by an efficient endogenous antioxidant system that identifies and alleviates the hazardous effects of oxidative metabolites, such as free radicals.^(13)^. [13]. Dysfunction of this defence system significantly contributes to the development of serious complications.^(14,15)^ A potent antioxidant’s primary function is to limit ROS-induced damage and maintain redox status as a cytoprotective mechanism.^(16)^

Supplemental natural antioxidants, such as polyphenols, vitamins, and terpenes, are mainly sourced from plants.^(17,18)^ There are two main categories of these natural bioactive compounds: the water-soluble (hydrophilic) group, which includes vitamin C, glutathione, and catechins; and the lipid-soluble (hydrophobic) group, which includes vitamins A and E.^(15)^

Hydrophilic antioxidants perform their activities in the cytoplasm and blood plasma, while hydrophobic antioxidants function in cellular membranes. The vast majority of conducted experimental and clinical studies clearly indicate the role of natural antioxidants in enhancing and maintaining vital bodily functions and slowing the signs of age-related changes.

Natural dietary antioxidants, including vitamins C and E, selenium, zinc, polyphenols, and carotenoids, are often lacking in regular food sources. This explains the growing demand for antioxidant supplements that support bioactive processes and maintain overall health. Potent supplementary antioxidants function by maintaining redox status and combating oxidation reactions.^(16–18)^

Cadmium (Cd) is one of the most hazardous toxic heavy metals that occurs naturally in the environment. It is used in a variety of industries. Exposure to cadmium can be occupational or non-occupational (environmental). Occupational exposure usually occurs through inhaling industrial fumes, while non-occupational exposure is often associated with ingesting contaminated food and water. Long-term cadmium accumulation results in chronic toxicity and progressive harmful effects in cells and tissues. Cadmium toxicity is associated with dramatic oxidative stress and tissue damage, particularly in the liver and kidneys.^(19)^

The usage of natural antioxidants as dietary supplements is on the rise as preventive and therapeutic strategies for various major health issues^(20,21)^, and many studies have focused on them. These beneficial effects have been observed in cardiovascular diseases, neurodegenerative disorders, cancer, arthritis, and age-related changes.^(22–24)^

The aim of this study was to evaluate the antioxidant properties of the catechin EGCG in male Wistar rats exposed to cadmium toxicity. The cadmium-induced oxidative stress represents the challenge for the tested antioxidant properties.

## Materials and methods

### Ethical considerations

The use and care of laboratory rats were strictly followed in accordance with institutional and national regulations (LAB-rats-2024-0379) and the Research Ethics Committee of Imam Mohammad Ibn Saud Islamic University (IMSIU). This animal study was performed in accordance with the following:

- UK guidance on the operation of the Animals (Scientific Procedures) Act of 1986 and associated guidelines

- EU Directive 2010/63 for the protection of animals used for scientific purposes

- The NIH (National Research Council) Guide for the Care and Use of Laboratory Animals

The study also complies with the ARRIVE (Animal Research: Reporting of In Vivo Experiments) guidelines.

### Type of sampling and reasons for selection

Whole blood, serum harvested from blood samples, and tissue homogenates were selected for investigation in this study. These samples were chosen to reflect changes in the haematological and biochemical profiles of the experimental rats.

### Inclusion and exclusion criteria

#### Inclusion criteria

The different experimental groups of rats were included in the conducted assays. Blood samples were collected from all rats for a haematological assay that assessed erythrocyte and leukocyte counts, haemoglobin concentration, and packed cell volume (PCV). Additionally, harvested serum samples and tissue homogenates from all rats were subjected to a biochemical assay to estimate antioxidant and oxidative parameter levels.

#### Exclusion criteria

No exclusion criteria were applied in this study. All experimental rats were included in the sample collection (blood and tissue). Any exclusion criteria could have altered the accuracy of the analysis.

### Experimental rats

For this experiment, we used 80 adult male Wistar rats, each three months old and weighing between 210 and 230 grams. The rats were obtained from inbred colonies at the animal facility of the College of Pharmacy at King Saud University in Riyadh, Saudi Arabia. They were maintained under standard laboratory conditions with a set ambient temperature of 24 ± 1°C, a 12-hour light-dark cycle, and a relative humidity level ranging from 35% to 70%.

### Catechin

#### Epigallocatechin gallate (EGCG)

(C_22_H_18_O_11_)

(−)-Cis-2-(3,4,5-trihydroxyphenyl)-3,4-dihydro-1(2H)-benzopyran-3, 5,7-triol, 3-gallate; (–)-cis-3,3′,4′,5,5′,7-hexahydroxyflavane-3-gallate; EGCG ≥ 80% (HPLC); CAS No.989-51-5, molecular weight 458.37), was purchased from Sigma-Aldrich (Darmstadt, Germany).

#### Cadmium

Cadmium (Cd) was used in the form of analytical-grade cadmium chloride (CdCl_2_; Merck, Darmstadt, Germany; product no. 655198). Cadmium chloride was dissolved in purified water to prepare the required aqueous solution.

### Experimental design

After a one-week acclimatisation period, the rats were randomly assigned to one of four groups of 20 rats each. The groups were designated Group 1, Group 2, Group 3, and Group 4. The rats in Group 1 served as the untreated control and were not exposed to cadmium or catechin. Group 2 rats received daily oral doses of catechin (ECGC) at 300 mg/kg body weight (BW) in a volume of 1 mL/kg BW.^(26–28)^ Group 3 rats were given an aqueous solution of cadmium chloride (CdCl_2_) via oral gavage at a final concentration of 5 mg/kg BW per day in a volume of 1 mL/kg BW. The control rats received an equal volume of saline via the same route. The rats in Group 4 received cadmium orally and also received catechin at the aforementioned doses. There was a 10-hour interval between the daily administration of cadmium and catechin.

The experimental period lasted eight weeks, during which the rats were provided with dry feed (commercial pellets from Envigo, USA) and drinking water ad libitum. All experimental rats were observed for behavioural activity, feed consumption, water intake, and clinical signs throughout the study.

### Haematological and biochemical assays

On the last day of the experiment, the rats were anaesthetised with 3% isoflurane. The euthanasia method complied with the American Veterinary Medical Association’s guidelines. Blood samples were collected via cardiac puncture from all the rats in the different groups. Blood samples collected with the anticoagulant EDTA were used to estimate various haematological indices. The serum was immediately harvested from the coagulated blood samples and stored at -20°C until biochemical assays were performed. The rats were then euthanised by decapitation. Their liver and kidney tissues were removed and homogenised in 150 mM NaCl. The homogenates were subsequently centrifuged at 3,000×g at 4°C for 10 minutes. The collected supernatants were used to determine various biochemical parameters.

#### Blood Cadmium Level

Blood cadmium levels were estimated by digesting 1 mL blood samples using a mixture of HClO_4_ and HNO_3_. The levels were then determined using an atomic absorption spectrophotometer (CBC 906 AA).

### Haematological assay

Various haematological parameters were measured using blood samples collected with the anticoagulant EDTA, including red blood cell counts, total white blood cell counts, and other erythrocytic indices, such as haemoglobin (Hb) concentration and PCV percentage. Erythrocyte and total leukocyte counts were measured using a haemocytometer. PCV percentage was determined using the microhematocrit method, and Hb concentration was estimated using the cyanmethemoglobin method, as previously described.

### Biochemical assay

The harvested serum from coagulated blood samples was used to estimate various biochemical parameters, including total proteins, albumin, globulin, creatinine, urea, blood urea nitrogen (BUN), alanine aminotransferase (ALT), aspartate aminotransferase (AST), and alkaline phosphatase (ALP). Antioxidant markers, such as total thiols, catalase, glutathione (GSH), superoxide dismutase (SOD), glutathione peroxidase (GSH-Px), and total antioxidant capacity (TAC), were also assessed. Oxidation markers, including malondialdehyde (MDA) and hydrogen peroxide (H_2_O_2_), were also measured.

Total thiols were measured using a colorimetric assay kit (Cell Biolabs Inc., USA) (MET-5053). Glutathione (GSH) levels were estimated using a reduced glutathione colorimetric assay kit (Elabscience, USA) (E-BC-K030-S). Catalase levels were determined using a catalase activity colorimetric assay kit (BioVision; Abcam, UK) (ab83464). Superoxide dismutase (SOD) activity was assessed using an SOD activity assay kit (Sigma-Aldrich, Darmstadt, Germany). Glutathione peroxidase activity was also measured using an Elabscience GSH-Px activity assay kit (Houston, Texas, USA).

Total antioxidant capacity (TAC) was estimated using a total antioxidant capacity assay kit (TAC assay kit) from Sigma-Aldrich (Germany) (MAK 187-1 KT). The kit measures the concentration of combined protein and small-molecule antioxidants or small-molecule antioxidants alone. During the process, Cu^2+^ ions are reduced to Cu^+^ by small molecules and proteins. However, the kit’s protein mask prevents proteins from reducing Cu²^+^, enabling the analysis of only small-molecule antioxidants. The reduced Cu^+^ ions produced by the small molecules are then chelated with a colorimetric probe. The resulting absorbance peak is directly proportional to the antioxidant capacity.

Hydrogen peroxide (H_2_O_2_) levels were measured using a colorimetric assay kit (Elabscience, USA) (E-BC-K102-S). MDA levels were assessed using an MDA colorimetric assay kit (Elabscience, USA) (E-BC-K028-M). ALT, AST, and ALP levels were measured using relevant diagnostic kits (Abcam, UK) (catalogue numbers: ALT ab105134, AST ab105135, and ALP ab83369).

Urea levels were determined using a colorimetric assay kit from BioVision (BioVision Incorporated, USA) with catalogue number K375-100. Blood urea nitrogen (BUN) levels were measured using a colorimetric BUN detection kit from ThermoFisher Scientific (catalogue no. EIABUN). Other biochemical parameters, including total proteins, creatinine, bilirubin, albumin, and globulin, were assessed using appropriate colorimetric diagnostic kits from Interchim Diagnostics (France). The corresponding catalogue numbers for these kits are as follows: total proteins (FT7250), creatinine (FT7040), bilirubin (FT6920), albumin (FT6760), and globulin (FT7253).

Tissue homogenates from the liver and kidney were prepared to measure total thiol, glutathione, catalase, hydrogen peroxide (H_2_O_2_), malondialdehyde (MDA), and total antioxidant capacity (TAC) levels in the tissues. The same assay kits were used to evaluate these parameters in the tissue homogenate serum.

### Statistical analysis

In the present study, the submitted data were expressed as means ± standard deviation (SD). Two-way ANOVA was conducted using SPSS software (SPSS Inc., Chicago, IL, USA) to compare the means among multiple groups. The normality and homogeneity of variances were verified, and independence of observations was confirmed. The normality of the data was verified using the Shapiro-Wilk test. Results with a p-value less than 0.05 (*P* < 0.05) were considered statistically significant.

## Results

Group 2 rats (administered with catechin) as well as those exposed to cadmium and also treated with catechin, displayed normal behaviour, activity levels, and food intake compared to the control rats that were not treated. In contrast, rats exposed to cadmium but not given catechin showed a decrease in activity and reduced food intake starting from the third week of the experiment, compared to the control group. Importantly, no deaths were observed among any of the rats in the experimental groups.

Rats of the control group, exhibited blood cadmium level at 0.0018 ± 0.0001 ppm. This level significantly increased (*P* < 0.05) in rats exposed to cadmium, reaching 0.577 ± 0.020 ppm. Notably, blood cadmium levels were lower in rats that were exposed to cadmium but also received catechin, with a measurement of 0.219 ± 0.017 ppm, compared to those exposed solely to cadmium.

Rats administered with catechin (Group 2) had no significant differences in haematological parameters compared to the control group. However, rats exposed to cadmium without catechin (Group 3) showed notable decreases in haemoglobin (Hb) concentration and PCV%. In contrast, rats exposed to cadmium and treated with catechin (Group 4) showed improvements in their haematological parameters, approaching control levels (Table 1).

**Table 1.** Haematological assay of rats that received catechin, exposed to cadmium, and that were exposed to cadmium and administered with catechin, compared to the control rats. RBC count, total leucocytic count, haemoglobin concentration, and packed cell volume percentage of cadmium-exposed rats were significantly lower compared to the control rats. The measured haematological indices in catechin-administered rats exhibited no significant differences compared to the control rats. In cadmium-exposed rats and those administered with catechin, the estimated haematological parameters showed significant increments compared to cadmium-exposed rats and were closer to the control levels

Parameter	Untreated control	Catechin	Cadmium	Cadmium and Catechin
**Total cell counts:**				
RBCs count (10^6^/mm^3^)	5.35 ± 0.11	5.51 ± 0.14	4.11* ± 0.15	5.46** ± 0.05
Total leucocytic Count (10^3^/mm^3)^	6.37 ± 0.38	6.29 ± 0.23	5.11* ± 0.27	6.17** ± 0.12
**Erythrocytic Indices:**				
Haemoglobin (Hb) concentration (g/dL)	12.39 ± 0.33	12.68 ± 0.29	9.57* ± 0.37	12.09** ± 0.41
Packed cell volume (PCV %)	44.19 ± 0.31	45.18 ± 0.29	36.26* ± 0.58	43.46** ± 0.81

Values are shown as means ± S.D., Number of rats/group =20.

*Significantly different means from that of untreated control rats (*P* < 0.05).

**Significantly different means from that of cadmium-exposed rats.

Table 1 shows the estimated haematological parameters in rats that received catechin, those exposed to cadmium, and those exposed to cadmium with catechin administration, compared to the control rats.

As to the biochemical profile, the rats that received catechin (Group 2) exhibited no significant changes in their biochemical parameters compared to the control group. In contrast, the rats in Group 3, which were exposed to cadmium and did not receive catechin, showed comparable decreases in the estimated levels of total proteins, albumin, and globulin. Furthermore, this group experienced a significant increase in creatinine, urea, and blood urea nitrogen (BUN) levels.

The levels of total thiols, glutathione, superoxide dismutase, glutathione peroxidase, and catalase were significantly lower in the serum and tissue homogenates of the group of rats exposed to cadmium that did not receive catechin (Group 3). Additionally, total antioxidant capacity (TAC) was significantly reduced in cadmium-exposed rats but significantly increased in those that received catechin.

The levels of malondialdehyde (MDA) and hydrogen peroxide (H_2_O_2_) in the serum and tissues of cadmium-exposed rats without access to catechin were significantly higher compared to the control levels (Table 2a, b, c).

**Table 2a.** Serum levels of total proteins, albumin, and globulin were significantly lower in cadmium-exposed compared to the untreated control rats. Levels of creatinine, urea, blood urea nitrogen (BUN), and bilirubin in the cadmium-exposed rats were significantly increased compared to the untreated control rats. Rats administered with catechin demonstrated no significant differences in their biochemical parameters compared to the untreated control rats. The estimated biochemical parameters in cadmium-exposed and catechin-treated rats exhibited improvements towards the control levels and were significantly different from those estimated in the cadmium-exposed rats

Parameter	Untreated control	Catechin	Cadmium	Cadmium and Catechin
Albumin (g/dL)	3.21 ± 0.07	3.31 ± 0.17	2.33* ± 0.15	3.11** ± 0.17
Globulin (g/dL)	3.79 ± 0.11	3.82 ± 0.18	2.34* ± 0.19	3.57** ± 0.35
Total proteins (g/dL)	7.45± 0.15	7.49 ± 0.25	5.29* ± 0.21	7.15** ± 0.15
**Liver function test:**				
Bilirubin (mg/dL)	6.31 ± 0.43	6.37 ± 0.37	11.47* ± 0.25	7.19** ± 0.51
**Kidney Function Test:**				
Creatinine (mg/dL)	0.54 ± 0.17	0.55 ± 0.16	0.89* ± 0.09	0.59** ± 0.32
Urea m(g/dL)	40.58 ± 0.46	40.62 ± 0.41	74.23* ± 0.63	48.29** ± 0.67
BUN (mg/dL)	15.29 ± 1.09	15.61 ± 1.07	29.39* ± 1.37	17.67 ± 1.49

Values are shown as means ± S.D., Number of rats/group =20.

*Significantly different means from that of untreated control rats (*P* < 0.05).

**Significantly different means from that of cadmium-exposed rats.

**Table 2b.** Serum levels of alanine transferase (ALT), aspartate transferase (AST), and alkaline phosphatase (ALP) in the different groups. The cadmium-exposed rats had significantly increased levels of all measured biochemical parameters. No significant differences were recorded in the catechin group compared to the control group. In the group of rats exposed to cadmium and administered with catechin, the biochemical parameters displayed improvements toward the control levels and were significantly decreased compared to the cadmium-exposed rats

Parameter	Untreated Control	Catechin	Cadmium	Cadmium and Catechin
**Serum enzymes**				
AST (IU/L)	41.21 ± 1.13	41.29 ± 1.09	139.27* ± 3.48	30.39** ± 1.11
ALT (IU/L)	26.49 ± 1.22	26.53 ± 1.13	69.17* ± 1.24	53.19** ± 1.19
ALP (IU/L)	25.21 ± 1.31	25.23 ± 1.17	75.65* ± 1.43	28.19** ± 1.61

Values are shown as means ± S.D., Number of rats/group =20.

*Significantly different means from that of untreated control rats (*P* < 0.05).

**Significantly different means from that of cadmium-exposed rats.

**Table 2c.** Serum levels of total thiols, glutathione (GSH), catalase, superoxide dismutase (SOD), glutathione peroxidase (GSH-Px), total antioxidant capacity (TAC), malondialdehyde (MDA), hydrogen peroxide (H_2_O_2_), and total antioxidant capacity (TAC) in the different groups. Compared to the untreated control rats, the cadmium-exposed rats exhibited significantly decreased levels of total thiols, GSH, catalase, and TAC. The levels of malondialdehyde (MDA) and hydrogen peroxide (H_2_O_2_) were significantly increased in cadmium-exposed rats compared to the untreated control rats. Catechin-treated rats showed no significant differences in the measured biochemical parameters compared to the untreated control rats. In the rats exposed to cadmium and treated with catechin, the biochemical parameters showed improvements toward the control levels and were significantly different compared to those of the cadmium-exposed rats

Parameter	Untreated Control	Catechin	Cadmium	Cadmium and Catechin
Oxidation Markers:				
MDA (nmol/mL)	310.11 ± 3.11	313.54 ± 3.18	445.27* ± 3.44	332.25** ± 3.57
H_2_O_2_ (nmol/mL)	40.63 ± 1.59	40.67 ± 1.51	90.17* ± 1.29	49. 13** ± 1.13
Antioxidant markers:				
Total thiols (mmol/L)	2.31 ± 0.29	2.32 ± 0.27	0.26* ± 0.11	2.07** ± 0.91
Glutathione (μg/mL)	40.81 ± 1.22	41.55 ± 1.12	13.89* ± 0.69	36.88** ± 1.41
Catalase (IU/L)	51.78 ± 1.61	52.68 ± 1.53	28.77* ± 1.11	46.71**± 1.27
SOD (U/mL)	6.33 ± 1.91	6.18 ± 1.66	3.38 ± 1.31	5. 55 ± 1.37
GSH-Px (U/mL)	137.03 ± 2.44	128.05 ± 2.21	76.02 ± 1.67	120.04 ± 1.68
TAC (nmol/mL)	35.39 ± 1.07	35.66 ± 1.19	16.69* ± 1.11	29.59** ± 1.17

Values are shown as means ± S.D., Number of rats/group = 20.

*Significantly different means from that of untreated control rats (*P* < 0.05).

**Significantly different means from that of cadmium-exposed rats.

Table 2a, b, c shows the biochemical parameters (serum levels) in rats that were administered catechin, those exposed to cadmium, and those exposed to cadmium and given catechin, compared to the control rats.

In rats that were exposed to cadmium and administered catechin (Group 4), the altered biochemical parameters estimated in tissue homogenates were brought closer to control levels (Table 3).

**Table 3.** Levels of total thiols, glutathione (GSH), catalase, superoxide dismutase (SOD), glutathione peroxidase (GSH-Px), total antioxidant capacity (TAC), malondialdehyde (MDA), hydrogen peroxide (H_2_O_2_), and total antioxidant capacity (TAC) in the tissue homogenates of different groups. The levels of total thiols, GSH, catalase, and TAC were significantly decreased in the tissue homogenates of cadmium-exposed rats compared to untreated control rats. MDA and H_2_O_2_ levels were significantly increased in the tissue homogenates of cadmium-exposed rats compared to untreated control rats. Catechin-treated rats showed no significant differences compared to untreated control rats. The estimated biochemical parameters in tissue homogenates of rats exposed to cadmium and administered with catechin demonstrated improvements toward the control levels and were significantly different compared to those measured in the cadmium-exposed rats

Parameter	Untreated Control		Catechin		Cadmium		Cadmium and Catechin	
	Liver	Kidney	Liver	Kidney	Liver	Kidney	Liver	Kidney
Catalase (IU/L) Total thiols (mmol/L) Glutathione (μg/mL) SOD (U/mL) GSH-Px (U/mL) MDA (nmol/mL) H_2_O_2_ (nmol/mL) TAC (nmol/mL)	20.19 ± 1.62 1.90 ± 0.31 16.50 ± 1.17 6.21 ± 1.81 131 ± 2.55 125.18 ± 3.34 14.89 ± 1.56 15.71 ± 1.24	19.17 ± 1.33 1.04 ± 0.29 14.71 ± 1.23 6.18 ± 1.81 126 ± 2.44 123.11 ± 3.79 14.81 ± 1.19 14.63 ± 1.17	19.83 ± 1.39 1.75 ± 0.27 16.57 ± 1.11 6.15 ± 1.73 130 ± 2.29 122.0 ± 13.22 16.46 ± 1.24 15.17 ± 1.11	18.85 ± 1.18 1.07 ± 0.29 14.74 ± 1.17 6.18 ± 1.55 113 ± 2.41 121.11 ± 3.22 14.39 ± 1.19 14.87 ± 1.17	9.16* ± 1.11 0.21* ± 0.18 5.06* ± 0.67 3.40 ± 1.63 80 ± 1.53 411.61* ± 3.41 88.90* ± 1.31 7.36* ± 1.14	9.13* ± 1.018 0.21* ± 0.17 4.97* ± 0.77 3.17 ± 1.67 75 ± 1.91 490.31* ± 3.71 97.11* ± 1.69 8.60* ± 1.13	17.71** ± 1.15 1.81** ± 0.36 14.70** ± 1.46 5.27 ± 1.93 117 ± 1.81 149.28** ± 3.19 18.41** ± 1.13 13.75** ± 1.17	17.43** ± 1.63 1.65** ± 0.57 14.81** ± 1.53 5.05 ± 1.69 112 ± 1.42 144.08** ± 3.74 19.20** ± 1.19 13.39** ± 1.11

Values are shown as means ± S.D., Number of rats/groups = 20.

*Significantly different means from that of untreated control rats (*P* < 0.05).

**Significantly different means from that of cadmium-exposed rats.

Table 3 shows the levels of total thiols, glutathione, catalase, glutathione peroxidase, superoxide dismutase, total antioxidant capacity (TAC), hydrogen peroxide (H_2_O_2_), and malondialdehyde (MDA) in the liver and kidney homogenates of rats that received catechin. Also shown are the measured levels in rats exposed to cadmium and those exposed to cadmium and administered catechin, compared to the control rats.

## Discussion

The current investigation used a catechin supplement with cadmium as an experimental model to evaluate antioxidant properties against induced oxidative stress. The study aimed to test whether catechin could improve haematological and biochemical alterations caused by cadmium-induced oxidative stress.

The endogenous antioxidant system comprises enzymes such as superoxide dismutase, catalase, and glutathione peroxidase, as well as non-enzymatic molecules. These antioxidants target specific free radicals and operate in different ways to mitigate their harmful effects, helping to maintain the balance between oxidative reactions and antioxidant activity. Antioxidants prevent chain reactions initiated by free radicals by stabilising these reactive molecules. They donate electrons to free radical molecules that possess unpaired electrons, thereby blocking their high reactivity.

Intra- and extracellular physiological metabolic processes, such as cell respiration, are mediated by free radicals. However, certain circumstances, including diseases and toxicities, generate excess amounts of free radicals.^(29)^ The body’s internal antioxidant system manages these excess free radicals to prevent their harmful effects.

Oxidative stress, also known as oxidative overload, occurs when there is an imbalance between oxidation reactions and antioxidant activities. This imbalance is referred to as redox status. This imbalance reduces the effectiveness of the body’s natural antioxidant system at managing the harmful effects of ROS. Disruptions to this balance caused by disease conditions or environmental factors are considered triggers of oxidative stress.^(30)^

Oxidative stress is characterised by the depletion of antioxidant molecules and plays a critical role in tissue damage and disease development. It is characterised by the excessive production of ROS, including free radicals such as hydroxyl and superoxide radicals, as well as non-radical compounds such as hydrogen peroxide.^(31)^

Free radicals have a hazardous impact when the antioxidant system is not efficient enough to counteract them. These unstable molecules can oxidise other cellular biomolecules, resulting in oxidative stress. This stress can damage cellular proteins and DNA, potentially leading to cell death. One significant outcome of this oxidative damage is lipid peroxidation, which primarily affects cell membranes.

Excessive generation of ROS is triggered by cadmium toxicity, which eventually leads to oxidative stress and significant damage to the liver and kidneys.^(32)^

Cadmium is toxic and leads to the oxidation of cell membrane lipids, significantly reducing adenosine triphospahte (ATP) production and glutathione levels in the mitochondria. Cadmium toxicity also disrupts the function of antioxidant enzymes, increasing oxidative stress. Ultimately, cadmium toxicity triggers apoptosis by activating caspases.^(33)^

Reliable markers used to assess antioxidant status in the present study include total thiols, glutathione, superoxide dismutase, glutathione peroxidase, catalase, and total antioxidant capacity (TAC), all of which were found to be significantly decreased in rats exposed to cadmium. These decreases suggest that excessive oxidative stress negatively impacted these antioxidants. It is proposed that ROS generated during this process caused the oxidation of these antioxidant molecules, leading to altered functions and reduced capacity to combat oxidative damage. Certain endogenous antioxidants, including glutathione and catalase, directly eliminate free radicals.

When antioxidants become oxidised and inactivated, free radicals increase, leading to heightened oxidative stress. Catalase breaks down hydrogen peroxide (H_2_O_2_), a compound strongly linked to lipid peroxidation. Reduced catalase activity allows hydrogen peroxide to exert powerful oxidative effects, particularly in rats. These effects contribute to the Fenton reaction, which produces highly damaging hydroxyl radicals (OH).^(34)^

The present results showed a significant increase in malondialdehyde (MDA) levels in cadmium-intoxicated rats, which could be attributed to lipid peroxidation of cell membranes. MDA is a biomarker of lipid peroxidation resulting from oxidative damage.^(35)^

The current biochemical profile indicates that cadmium toxicity substantially affects the body’s natural antioxidant system and alters the blood’s biochemical profile. This has led to a depletion of antioxidant molecules, decreased overall antioxidant capacity, and disrupted the natural balance between oxidants and antioxidants. When the body’s natural antioxidant system is compromised, introducing external sources of antioxidants becomes essential to restore this balance. Antioxidants in supplement form are expected to protect biological systems from oxidative damage by quickly eliminating excess free radicals and enhancing the body’s antioxidant system. Supplemented and endogenous antioxidants collaborate to neutralise and eliminate free radicals. It is worth mentioning that the byproducts resulting from the reaction of antioxidant molecules with free radicals can eliminate additional radicals, thus enhancing the overall antioxidant effectiveness.^(35,36)^

Serum enzymes (ALT, AST, and ALP) were significantly elevated in cadmium-exposed rats, indicating liver damage induced by cadmium. This increase may be attributed to lysosomal damage caused by lipid peroxidation, which releases enzymes into the bloodstream and leads to higher enzyme levels.

The levels of these serum enzymes, which are linked to hepatic function, were not elevated in rats administered catechins compared to the control group. This excludes the possibility of a hepatotoxic effect from the catechin dose used. Furthermore, cadmium exposure resulted in increased blood urea and creatinine levels, suggesting kidney damage in the affected rats. Blood urea nitrogen (BUN), urea, and creatinine levels were not elevated in the catechin-administered rats compared to the control group. This excludes any potential nephrotoxic effects of the utilised catechin dose.

Catechins are not synthesised in the human body, but their bioactivities make them representative of exogenous antioxidants, which enhance the activity of the antioxidant system and combat oxidative stress. Previous studies have examined catechin’s potential to combat oxidative stress and related damage while maintaining redox status.^(37)^

The potent antioxidant properties of catechins are mainly related to the hydroxyl groups in their chemical structure. The location and number of these groups on the aromatic ring of epigallocatechin gallate (EGCG) explain its greater antioxidant efficacy compared to other catechins.^(38–40)^ ECGC’s ability to combat ROS is attributed to the structure of the D- and B-rings.^(41,42)^ Catechins’ antioxidant properties are explained by multiple mechanistic pathways, including the scavenging of free radicals (such as peroxyl radicals, superoxide anions, nitric oxide, peroxynitrite, and hydrogen peroxide), the neutralisation of radicals, and the inhibition of lipid peroxidation.^(43,44)^ Two mechanisms explain the antioxidant activities of catechins: the hydrogen atom transfer mechanism and the single-electron transfer mechanism. In the first mechanism, free radicals receive a hydrogen atom from antioxidants and become more stable products. In the second mechanism, antioxidants donate a single electron to free radicals, which then become stabilised.^(45,46)^ Furthermore, ECGC modulates signalling pathways (NF-κB, PKC, MAPK, Nrf2, and PI3K/Akt), which are disrupted by oxidative stress. In this way, ECGC enables the antioxidant system to eliminate ROS. Catechins, especially ECGC, increase the activities of SOD, catalase, and GPX through the inhibitory modulation of Nrf2, which downregulates the expression of antioxidant enzymes.^(47,48)^

Catechins, especially epigallocatechin gallate (EGCG), increase the activities of superoxide dismutase (SOD), catalase, and glutathione peroxidase (GPX) by inhibiting the modulation of nuclear factor erythroid 2-related factor 2 (Nrf2), which downregulates the expression of antioxidant enzymes. ECGC has been demonstrated to ameliorate excess NO levels, which, under conditions of oxidative stress, can induce RN (reactive nitrogen species) generation in the form of peroxynitrite.^(45,46)^

ECGC can also inhibit xanthine oxidase activity, thereby mitigating increasing levels of ROS.^(49,50)^

Significantly decreased blood cadmium levels in rats previously intoxicated with cadmium and then treated with catechin are attributed to cadmium chelation. Flavonoids possess the property to chelate metals to form metal complexes, and this help counter the activity of the oxidising agents through inhibiting the liberation of free iron that contributes to Fenton reaction.^(51,52)^ ECGC can chelate transition metals^(53)^, and this metal-chelating property is related to the ortho-3,4-dihydroxy and 4-keto,3-hydroxyl moieties.^(42)^ The metal chelating efficacy of catechin was found to be comparable to that of potent flavonoids such as quercetin.^(54)^

Undoubtedly, reducing cadmium levels in the blood significantly limits the initiative step of oxidative stress and contributes to the recovery of the endogenous antioxidant system.

The present findings align with previous studies that concluded catechins enhance the antioxidant system. As demonstrated in this study, epigallocatechin gallate (ECGC) has the potential to significantly increase the expression of antioxidant enzymes, which are reliable biomarkers of antioxidant activity.^(55)^

Additionally, the present results provide evidence that epigallocatechin gallate (EGCG) effectively inhibits lipid peroxidation, as indicated by the significant decrease in malondialdehyde (MDA) levels, a marker of lipid peroxidation.^(56,57)^

In brief, the present experimental work demonstrated significant increases in antioxidant markers, including total thiols, glutathione, catalase, glutathione peroxidase, superoxide dismutase, and total antioxidant capacity, in rats exposed to cadmium and administered ECGC catechin. These results support the positive impact of ECGC on the expression of antioxidant molecules and highlight its role in enhancing endogenous antioxidant capacity.

Haematological and biochemical assays conducted on rats exposed to cadmium and treated with ECGC exhibited improved parameters that were restored to control levels. These results may support the hypothesis that catechins have a synergistic antioxidant role in reducing oxidative stress induced by cadmium toxicity.

The results clearly demonstrate ECGC’s antioxidant properties in reducing oxidative stress and damage caused by cadmium toxicity. However, further research is necessary to fully understand the molecular mechanisms by which this potent catechin exerts its antioxidant effects in the context of heavy metal toxicity. In other words, while these results demonstrate the effectiveness of ECGC as an antioxidant, additional research is necessary to elucidate the detailed molecular mechanisms underlying these properties.

## Conclusion

Based on the assessment and analysis of current haematological and biochemical assays, it can be concluded that the catechin epigallocatechin gallate (ECGC) possesses antioxidant potential to counteract cadmium-induced oxidative stress and restore the oxidation-antioxidation balance.

## Data Availability

Data will be made available on request.

## Abbreviations:

-EGCG (epigallocatechin-3-Gallate)

-ROS (reactive oxygen species)

-ALT (alanine transferase)

-AST (aspartate transferase)

-ALP (alkaline phosphatase)

-GSH (glutathione)

-SOD (superoxide dismutase)

-GSH-Px (glutathione peroxidase)

-TAC (total antioxidant capacity)

-MDA (malondialdehyde) and

-TAC (total antioxidant capacity)
